# Iteratively refining breast cancer intrinsic subtypes in the METABRIC dataset

**DOI:** 10.1186/s13040-015-0078-9

**Published:** 2016-01-13

**Authors:** Heloisa H. Milioli, Renato Vimieiro, Inna Tishchenko, Carlos Riveros, Regina Berretta, Pablo Moscato

**Affiliations:** Priority Research Centre for Bioinformatics, Biomarker Discovery and Information-Based Medicine, Hunter Medical Research Institute, Lot 1, Kookaburra Circuit, New Lambton Heights, 2305 Australia; School of Environmental and Life Science, The University of Newcastle, University Drive, Callaghan, 2308 Australia; Centro de Informática, Universidade Federal de Pernambuco, Av. Prof. Moraes Rego, Recife, Brazil; School of Electrical Engineering and Computer Science, The University of Newcastle, University Drive, Callaghan, 2308 Australia

**Keywords:** Breast cancer, Intrinsic subtypes, Predictor models, Subtype prediction, METABRIC, CM1 score, Feature selection, Data mining, Ensemble learning, Classifiers

## Abstract

**Background:**

Multi-gene lists and single sample predictor models have been currently used to reduce the multidimensional complexity of breast cancers, and to identify intrinsic subtypes. The perceived inability of some models to deal with the challenges of processing high-dimensional data, however, limits the accurate characterisation of these subtypes. Towards the development of robust strategies, we designed an iterative approach to consistently discriminate intrinsic subtypes and improve class prediction in the METABRIC dataset.

**Findings:**

In this study, we employed the CM1 score to identify the most discriminative probes for each group, and an ensemble learning technique to assess the ability of these probes on assigning subtype labels using 24 different classifiers. Our analysis is comprised of an iterative computation of these methods and statistical measures performed on a set of over 2000 samples. The refined labels assigned using this iterative approach revealed to be more consistent and in better agreement with clinicopathological markers and patients’ overall survival than those originally provided by the PAM50 method.

**Conclusions:**

The assignment of intrinsic subtypes has a significant impact in translational research for both understanding and managing breast cancer. The refined labelling, therefore, provides more accurate and reliable information by improving the source of fundamental science prior to clinical applications in medicine.

**Electronic supplementary material:**

The online version of this article (doi:10.1186/s13040-015-0078-9) contains supplementary material, which is available to authorized users.

## Findings

Translational research aims at bringing basic scientific discoveries into outcomes that help improve clinical decision-making. The PAM50 Breast Cancer Intrinsic Classifier [1] has lately been used to assign the molecular subtypes (luminal A, luminal B, HER2-enriched, basal-like and normal-like [2–5]) based on shrunken centroids of gene expression profiles [6]. It uses a Single Sample Predictor (SSP) model with an embedded 50-gene assay. In spite of the relevance of this method for clinical management, there are limited investigations in the literature that support this classification approach. Comparison with other methods showed only moderate agreement between subtype labels assigned, as well as independent clinical prognostic information [7–9].

Other multi-gene signatures have also been reported within the molecular patterns strongly correlated to clinical prognosis [10, 11], disease progression [12, 13], and patient survival [14]. Different methods, however, highlight a variety of gene lists of distinct size due the analysis of diverse microarray data and platform technologies. Additionally, the methods currently applied bring a pragmatic concern of using SSP models for predicting disease subtypes. Multiple classifiers or ensemble learning model, on the other hand, have compensated for poor learning algorithms by performing extra computation [15]. Therefore, there is an urgent need for translating these novel strategy to provide more accurate predictions of clinicopathological outcome.

In 2012, The Molecular Taxonomy of Breast Cancer International Consortium (METABRIC) [16] disclosed a rich gene expression cohort widely used for investigating breast cancer diseases. In spite of the quality of this dataset, there are some inconsistencies with regards to the subtype labels assigned in the original cohort [17]. In our previous study [17], a thorough review of the intrinsic subtypes was suggested and is, therefore, mandatory given the importance of this dataset to breast cancer research. For this report, we then propose a more robust approach to iteratively refine the labels in the METABRIC dataset based on ensemble learning. The new labels are yet correlated to well-established clinicopathological markers and patient overall survival.

## Methods

### Transcriptomic datasets

The breast cancer dataset disclosed by the METABRIC study (EGAS00000000083) contains cDNA microarray profiling of about 2000 samples performed on the Illumina HT-12 v3 platform (Illumina_Human_WG-v3) [16]. The samples were originally partitioned into two subsets: *Discovery* (997 samples) and *Validation* (989 samples), respectively used as *training* and *test* sets in our analysis. In this cohort, tumour samples were assigned on the five intrinsic subtypes (luminal A, luminal B, HER2-enriched, basal-like and normal-like) according to the PAM50 method [1]. The METABRIC study was approved by the Institutional Review Board [16] and our research was authorized by the Human Ethics Research Committee at The University of Newcastle, Australia (H-2013-0277).

### Refinement method

**Fig. 1:**
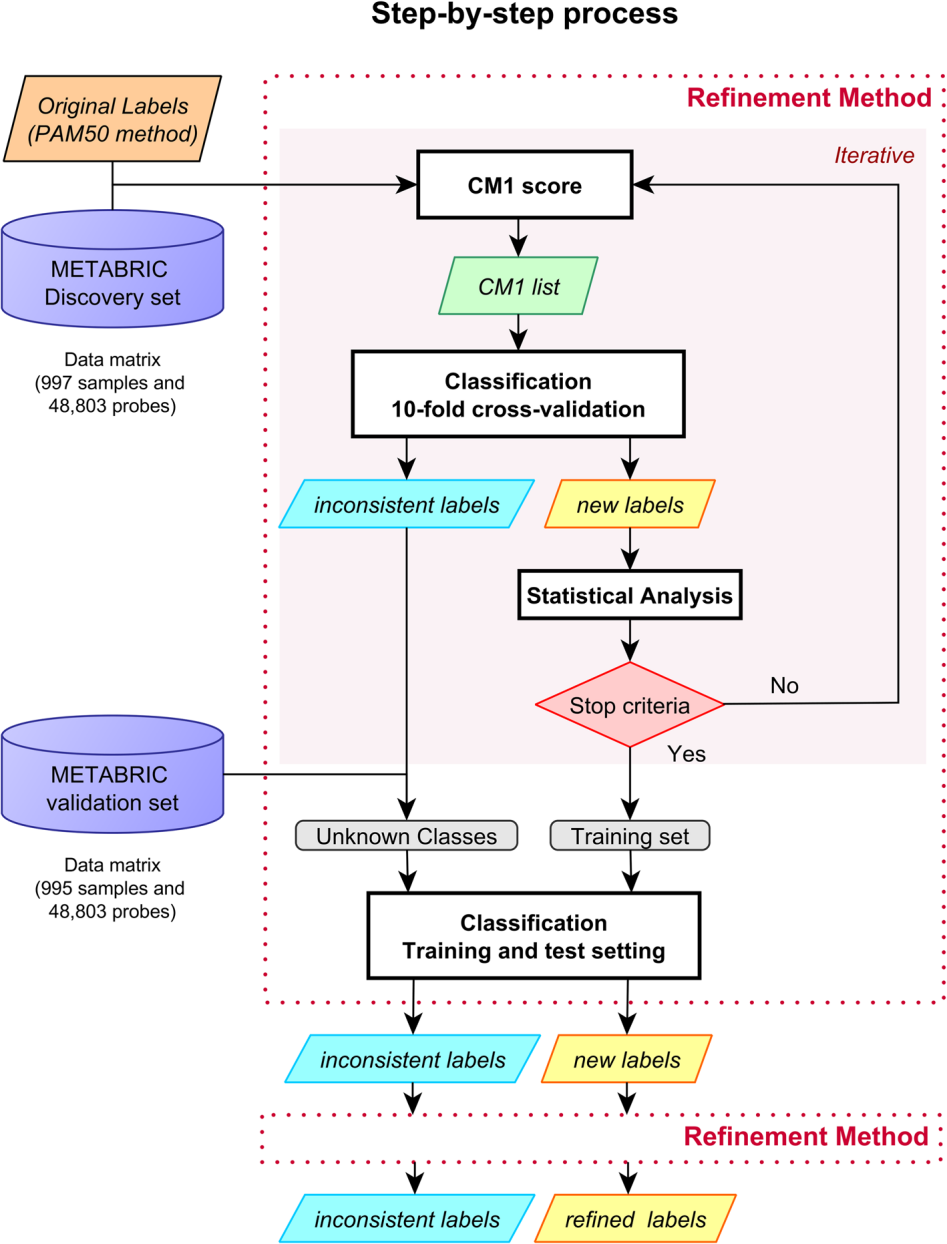
Refinement process. The process is initialized with labels assigned using the PAM50 method. After computing the CM1 score, the top 10 highly discriminative probes are selected for each subtype. This set of features is used to train the 24 distinct classifiers for a 10-fold cross-validation classification. Samples are relabelled (eventually with the same label) if the classifiers agree in at least 50 % of the cases; otherwise they are marked as inconsistent and not further considered in the iteration process. The stopping criterion is reached when there are no more changes in the sample labels or selected feature set, or when the desired Fleiss’ kappa is achieved. After stopping, the final feature set and sample labels are used to classify the samples previously marked as inconsistent or from the validation dataset. These samples are run through the same refinement procedure; inconsistent samples are reclassified and labels are refined

The overview of the refinement method applied on the METABRIC dataset is shown in Fig. 1. The process is initialized with the discovery set and the original PAM50 labels as defined in Curtis et al. [16]. After computing the CM1 score, the top ten highly discriminative probes (five with the greatest positive CM1 score values - indicating up-regulated probes relative to the other subtypes, and five with the smallest negative values – representing down-regulation) are chosen for each class. The set of new features is used to train the 24 classifiers from the Weka software suite [18], where a ten-fold cross-validation is performed. If the majority of the classifiers agree on the same label, the sample is assigned with the corresponding subtype; otherwise it is marked as inconsistent and not further considered in the process. The stopping criterion is reached when there are no more changes in the sample labels and feature set, or when the desired Fleiss’ kappa value (*κ*≥0.92) is achieved between the previous and the current iteration steps (see Section Statistics below for definitions). Values between 0.81 and one are considered to be *almost perfect agreement*, thus 0.92 is above the average for this interval.

When the stopping condition is fulfilled, the new list of features and sample labels are used for the training-test setting. Samples from the validation dataset or previously marked as inconsistent are then classified by training the classifiers in the refined discovery set. However, in the training-test setting, at least two thirds of classifiers in the ensemble must agree on the same label for it to be assigned to a sample. As a larger dataset is expected to provide more robustness, all the reclassified samples are run through the same refinement procedure again. The final outcome of this process is the set of refined features and the new labels.

Since many classifiers tend to perform best when trained on classes of equal sample size, we adjusted the number of patients in each subtype by looking at the minimum number of samples in one of the subgroups. The normal-like subtype is represented by only 58 samples; thus, the total number of samples used in the training is 290. For each other subtype, 58 samples are randomly chosen from the dataset. The whole process is run ten times due to the interchangeable sample selection that weigh the different gene expression information used for training purposes.

### The CM1 score

The CM1 score is a supervised method used to rank the variation of gene expression levels across samples from two different classes (more details in [17, 19]). The measure helps to identify the most discriminative features for each of the five breast cancer intrinsic subtypes: luminal A, luminal B, HER2-enriched, basal-like and normal-like. For a given subtype, we compute the CM1 score for each of the 48,803 probes and select the ten most discriminative ones. This happens iteratively in the refinement process each time the classifiers attribute a new label to a sample.

### Statistics

Several measures have been computed in order to assess the quality of our results. We created a contingency table *r*×*c* comparing the predicted labels (rows) and labels from the previous refinement step (columns).

*Cramer’s V* [20] is used to measure the level of association between sample original and predicted labels. The statistic ranges from zero (no association between the two variables) to one (complete association).

*Fleiss’ kappa* [21, 22] is a popular interrater reliability metric used to gauge the agreement between the original PAM50 labels and the labels assigned by the majority of classifiers. Kappa values range from ≤0 to 1, where: (1) values ≤0 show a *poor agreement*; (2) 0≤*κ*≤0.2, *slight agreement*; (3) 0.21≤*κ*≤0.40, *fair agreement*; (4) 0.41≤*κ*≤0.60, *moderate agreement*; (5) 0.61≤*κ*≤0.80*substantial agreement*; and (6) 0.81≤*κ*≤1, *almost perfect agreement*.

*Adjusted Rand Index (ARI)* [23, 24] measures the agreement between pairs of samples that are labelled either in the same class or in different classes. Results range from zero (complete discordance between two partitions) to one (perfect concordance between them).

### Clinical data and survival analysis

The clinical markers oestrogen and progesterone receptors (ER and PR) and the human epidermal growth factor receptor two (*HER2*) are compared between original METABRIC labels and refined labels. Survival analysis was also performed, using Cox proportional hazards model from the package *survival* in the R software [25]. The *p*-value, used to test the null hypothesis that the curves stratified by subtype are identical in the overall population, is calculated using the log-rank test.

## Results and Discussion

### Discriminative probes used to assign the intrinsic subtype labels in the refinement process

**Fig. 2:**
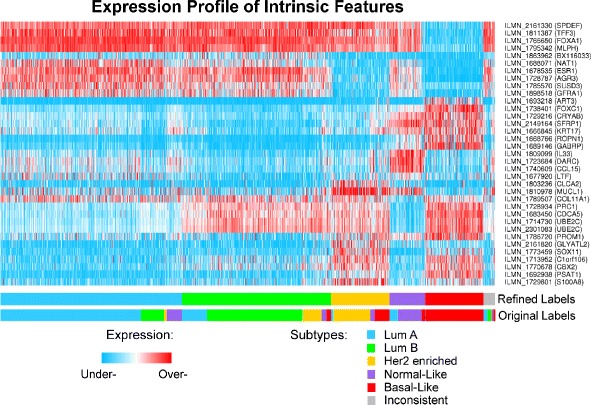
The heat map of refined intrinsic features selected using CM1 score in the refinement process. The heat map diagram exhibit 35 probes (rows) and 1992 samples (columns) from the discovery and validation sets ordered according to gene expression similarity. For visualisation, the expression values are normalised across the probes using a two-sided threshold of 1 % (for under- and over-expression). The bars on the bottom show the sample distribution according to the refined and original labels assigned to the METABRIC cohort. The subtypes are defined as follow: luminal A (blue), luminal B (green), HER2-enriched (yellow), normal-like (purple), basal-like (red), and inconsistent (grey)

Samples were assigned into the five intrinsic subtypes based on the majority voting of classifiers (Additional file 1: Table S1), supported by their consistent performance across the ten runs (Additional file 2). During this procedure, 74 discriminative probes appeared (Additional file 1: Table S2) and, among them, 35 were recurrently selected (Fig. 2). Overall, the association between the initial labels and those predicted using the ensemble learning (Table 1) was 0.95 according to Cramer’s V. The consensus of sample labelling across different classifiers measured using Fleiss’ kappa was 0.924. The ARI (1.00) also showed a maximum agreement between pairs of samples that are labelled either in the same class or in different classes.

**Table 1:** Contingency table for predicted labels vs. initial subtypes (rows and columns, respectively)

Subtypes	Lum A	Lum B	HER2	Basal	Normal	Summary
Lum A	563	94	11	2	58	728
Lum B	102	383	77	19	19	600
HER2	7	1	149	59	18	234
Basal	0	0	0	230	3	233
Normal	33	0	1	15	95	144
Inconsistent	16	14	2	6	9	47
Summary	721	492	240	331	202	1986

### New subtype labels reveal more reliable distribution of clinical markers and survival curves

We correlated the METABRIC and predicted labels with the current clinical markers ER, PR and *HER2*. Table 2 shows the changes in number of samples across subtypes, labelled with the PAM50 method and refined labels, respectively. The refinement process improved the overall distribution to what is expected for each class: luminal A (ER+, PR+, *HER2*–), luminal B (ER+, PR ±, *HER2* ±), HER2-enriched (ER–, PR–, *HER2*+) and basal-like (ER–, PR–, *HER2*–); especially for HER2-enriched and basal-like subtypes. Samples labelled as inconsistent in our study may also reflect the heterogeneity of the disease and a hint to as-yet improperly characterized molecular subtypes.

**Table 2:** Number of samples for each clinical marker in the PAM50 subtypes and refined labels

*PAM50 subtypes*						
Class ∖Marker	PR+	PR-	ER+	ER-	HER2+	HER2-
Luminal A	550	171	717	4	23	698
Luminal B	309	183	492	0	45	447
Her2-enriched	51	189	98	142	135	105
Basal-like	29	302	41	290	30	301
Normal-like	106	96	164	38	16	186
*Refined labels*						
Class ∖Marker	PR+	PR-	ER+	ER-	HER2+	HER2-
Luminal A	558	170	726	2	14	714
Luminal B	358	242	599	1	83	517
Her2-enriched	11	223	19	215	139	95
Basal-like	7	226	9	224	4	229
Normal-like	85	59	115	29	4	140
Inconsistent	26	21	44	3	5	42

**Fig. 3:**
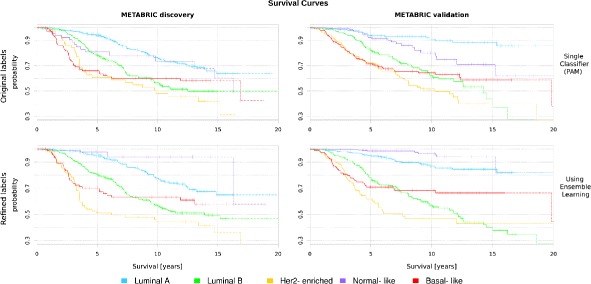
The survival curves for original and refined labels in the METABRIC discovery and validation sets. Each curve represents the survival probability at a certain time after the diagnosis. Drops in the curve indicate death. The probability of the last ten observations are plotted in dash

Furthermore, the patient’s overall survival significantly improved across subtypes when the original and refined labels are used to plot the curves for the METABRIC discovery and validation sets (Fig. 3). The groups have a well defined separation after the refinement process (*p* value 2.8×10^−26^) compared to the original labels (*p* value 5.4×10^−18^). These results also support a better characterization of the intrgroups after the iterative approach.

## Conclusion

The iterative approach using CM1 score and ensemble learning has shown a great potential for predicting more accurate sample subtypes in the METABRIC breast cancer dataset. The refined labels are of great value to breast cancer research and future clinical translational science. Given the relevance of accurate subtype assignments, we encourage researchers to consider the proposed refined labels when analysing the METABRIC dataset.

## Additional files

Additional file 1
**Refined subtype labels and intrinsic probes.** The refined breast cancer subtype labels defined for each sample in the METABRIC dataset are listed in Table S1. Table S2 shows the annotated probes selected in the CM1 list and the average occurrence of each probe. (XLSX 58 kb)

Additional file 2
**Classifiers Performance.** The document contains information on the ensemble learning approach with regards to the performance of each classifier. (PDF 1280 kb)

## Abbreviations

ARI: adjusted rand index
EGA: european genome-phenome archive
ER: oestrogen receptor
HER2: human epidermal growth factor receptor 2
METABRIC: molecular taxonomy of breast cancer international consortium
PR: progesterone receptor
SSP: single sample predictor
