# Supplementary material for: Iteratively refining breast cancer intrinsic subtypes in the METABRIC dataset
Source: BioData Min. 2016 Jan 13;9:2. doi: 10.1186/s13040-015-0078-9 (PMC4712506; doi:10.1186/s13040-015-0078-9)
Supplement: Additional file 2 — Classifiers Performance. The document contains information on the ensemble learning approach with regards to the performance of each classifier. (PDF 1280 kb) [file 13040_2015_78_MOESM2_ESM.pdf]

Classifiers Performance

Additional File 2

November 2015

Information on the classifiers used in the manuscript: *Iteratively refining breast cancer intrinsic subtypes in the METABRIC dataset.*

The idea of an ensemble learning approach is to obtain better predictive performance than could be obtained from any of the constituent learning algorithms ([18, 20]). In statistics and machine learning, ensemble methods use multiple learning algorithms that typically allows for a more flexible structure to exist among individual alternatives. Evaluating the prediction of an ensemble requires more computation than evaluating the prediction of a single models; as a way to compensate for poor learning algorithms. The majority vote to assign the sample subtype is, accordingly, more reliable than the average of the classifiers, for example.

The set of classifiers used in this work correspond to a diverse group of classifier families, as implemented in the Weka 3.7.12 software package [9]. The list of classifiers are given in Table 1. Classifiers are used with their default values, and experiments are repeated 10 times with different random seeds to provide an estimate of true value. The average mean performance of each classifier is shown in Figure 1. As can be observed, all classifiers attain a Kappa value greater than 0.89, which is considered an *almost perfect agreement* [21]. The average agreement per subtype is also presented in Table 2.

Moreover, during the course of the refinement iterations, agreement among classifiers increases significantly, and more importantly, in a consistent manner. The evolution of the agreement, as measured by κ versus the final set of labels, for a typical iteration run is shown in Figure 2.

Table 1: List of the 24 classifiers used in the ensemble learning

**Classifier**	**Family**	**Software Author**	**Reference**
BayesNet	bayes	Remco Bouckaert	
NaiveBayes	bayes	Len Trigg, Eibe Frank	[12]
NaiveBayesUpdateable	bayes	Len Trigg, Eibe Frank	[12]
Logistic	functions	Xin Xu	[16]
MultilayerPerceptron	functions	Malcolm Ware	
SimpleLogistic	functions	Niels Landwehr, Marc Sumner	[15, 22]
SMO	functions	Eibe Frank, Shane Legg, Stuart Inglis	[17, 10, 13]
IBk	lazy	Stuart Inglis, Len Trigg, Eibe Frank	[1]
KStar	lazy	Len Trigg, Abdelaziz Mahoui	[4]
AttributeSelectedClassifier	meta	Mark Hall	
Bagging	meta	Eibe Frank, Len Trigg, Richard Kirkby	[2]
ClassificationViaRegression	meta	Eibe Frank, Len Trigg	[7]
LogitBoost	meta	Len Trigg, Eibe Frank	[8]
MultiClassClassifier	meta	Eibe Frank, Len Trigg, Richard Kirkby	
RandomCommittee	meta	Eibe Frank	
DecisionTable	rules	Mark Hall	[14]
JRip	rules	Xin Xu, Eibe Frank	[5]
PART	rules	Eibe Frank	[6]
HoeffdingTree	trees	Richard Kirkby, Mark Hall	[11]
J48	trees	Eibe Frank	[19]
LMT	trees	Niels Landwehr, Marc Sumner	[15, 22]
RandomForest	trees	Richard Kirkby	[3]
RandomTree	trees	Eibe Frank, Richard Kirkby	
REPTree	trees	Eibe Frank	

The family and implementation authors are given. The **Reference** *is the source of the method algorithm, when available.*

Table 2: Average agreement of classifiers per subtype

**Subtypes**	**Agreement**	**Agreement (no Inc.)**
Luminal A	0.8375	0.8962
Luminal B	0.8762	0.918
HER2-enriched	0.9415	0.9926
Basal-like	0.9567	0.9906
Normal-like	0.7896	0.9024
**Average**	**0.8803**	**0.93996**

The numbers represent the average agreement calculated across ten runs, with relation to the final labels. The "no Inc", in the second column, excludes samples labelled "Inconsistent" from the calculation, while in the first column all samples are taken.

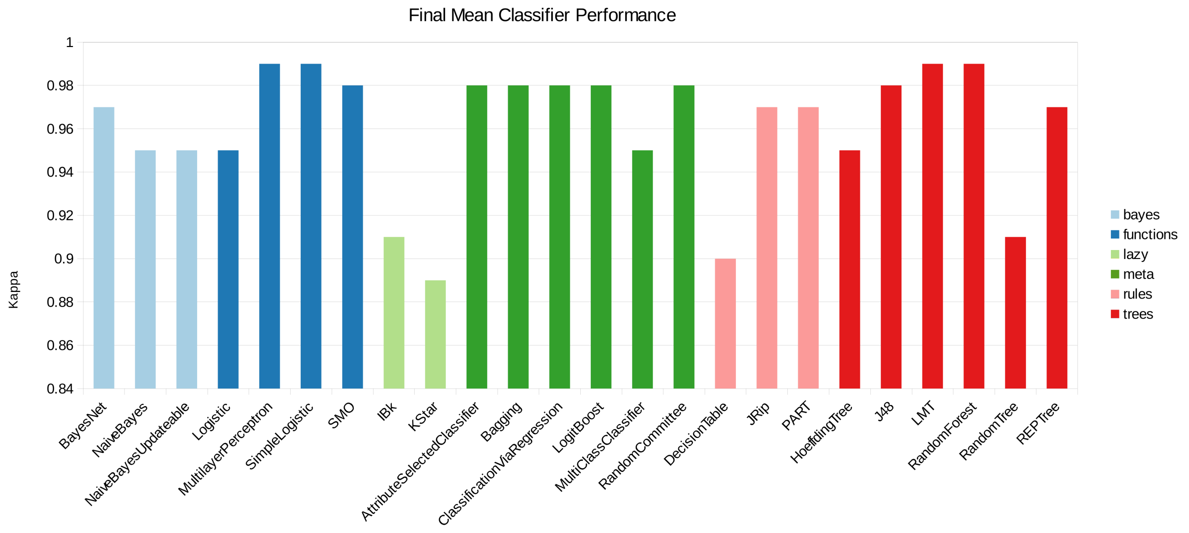

Figure 1: Mean Final Classifier Performance, as measured by Fleriss' κ against the final ensemble learning labels of all samples, across the 10 different refinement runs

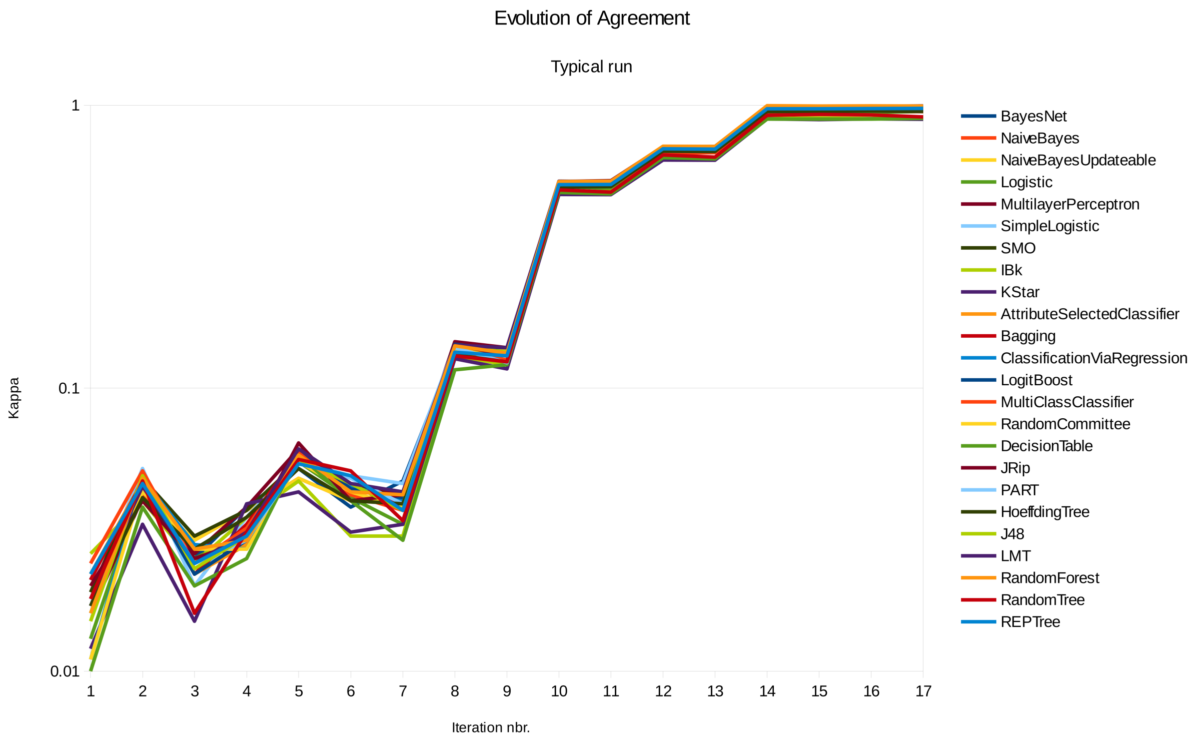

Figure 2: Evolution of performance of classifiers along iterations in a typical refinement run. κ values are measured against final ensemble learning labels.

References

[1] David W. Aha, Dennis Kibler, and Marc K. Albert. "Instance-based learning algorithms". In: *Machine Learning* 6.1 (1991), pp. 37–66. ISSN: 1573-0565. DOI: 10.1007/bf00153759.

[2] Leo Breiman. "Bagging predictors". In: *Machine Learning* 24.2 (1996), pp. 123–140. ISSN: 1573-0565. DOI: 10.1007/bf00058655.

[3] Leo Breiman. "Random forests". In: *Machine learning* 45.1 (2001), pp. 5–32. ISSN: 0885-6125. DOI: 10.1023/A:1010933404324.

[4] John G Cleary and Leonard E Trigg. "K*: An instance-based learner using an entropic distance measure". In: *Proceedings of the 12th International Conference on Machine learning*. Vol. 5. 1995, pp. 108–114.

[5] William W Cohen. "Fast effective rule induction". In: *Proceedings of the twelfth international conference on machine learning*. 1995, pp. 115–123.

[6] Eibe Frank and Ian H Witten. "Generating accurate rule sets without global optimization". In: *Computer Science Working Papers, University of Waikato, Department of Computer Science* 98/2 (1998).

[7] Eibe Frank et al. "Using model trees for classification". In: *Machine Learning* 32.1 (1998), pp. 63–76.

[8] Jerome Friedman, Trevor Hastie, and Robert Tibshirani. "Additive logistic regression: a statistical view of boosting (with discussion and a rejoinder by the authors)". In: *The annals of statistics* 28.2 (2000), pp. 337–407.

[9] Mark Hall et al. "The WEKA data mining software: an update". In: *SIGKDD Explor. Newsl.* 11.1 (Nov. 2009). ACM ID: 1656278, pp. 10–18. ISSN: 1931-0145. DOI: 10.1145/1656274.1656278.

[10] Trevor Hastie and Robert Tibshirani. "Classification by Pairwise Coupling". In: *The annals of statistics* 26.2 (1998). Ed. by Michael I. Jordan, Michael J. Kearns, and Sara A. Solla, pp. 451–471.

[11] Geoff Hulten, Laurie Spencer, and Pedro Domingos. "Mining time-changing data streams". In: *Proceedings of the seventh ACM SIGKDD international conference on Knowledge discovery and data mining*. ACM. 2001, pp. 97–106.

[12] George H John and Pat Langley. "Estimating continuous distributions in Bayesian classifiers". In: *Proceedings of the Eleventh conference on Uncertainty in artificial intelligence*. Morgan Kaufmann Publishers Inc. 1995, pp. 338–345.

[13] S. Sathiya Keerthi et al. "Improvements to Platt's SMO algorithm for SVM classifier design". In: *Neural Computation* 13.3 (2001), pp. 637–649.

[14] Ron Kohavi. "The power of decision tables". In: *Machine Learning: ECML-95*. Springer, 1995, pp. 174–189.

[15] Niels Landwehr, Mark Hall, and Eibe Frank. "Logistic model trees". In: *Machine Learning* 59.1-2 (2005), pp. 161–205.

[16] Saskia Le Cessie and Johannes C Van Houwelingen. "Ridge estimators in logistic regression". In: *Applied statistics* 41.1 (1992), pp. 191–201. DOI: 10.2307/2347628.

[17] John C Platt. "Fast Training of Support Vector Machines Using Sequential Minimal Optimization". In: *Advances in Kernel Methods - Support Vector Learning.* MIT Press, 1998.

[18] Robi Polikar. "Ensemble based systems in decision making". In: *Circuits and Systems Magazine, IEEE* 6.3 (2006), pp. 21–45. ISSN: 1531-636X. DOI: 10.1109/MCAS.2006.1688199.

[19] J Ross Quinlan. *C4. 5: programs for machine learning.* Elsevier, 2014.

[20] Lior Rokach. "Ensemble-based classifiers". English. In: *Artificial Intelligence Review* 33.1-2 (2010), pp. 1–39. ISSN: 0269-2821. DOI: 10.1007/s10462-009-9124-7.

[21] Julius Sim and Chris C Wright. "The kappa statistic in reliability studies: use, interpretation, and sample size requirements". In: *Physical therapy* 85.3 (2005), pp. 257–268.

[22] Marc Sumner, Eibe Frank, and Mark Hall. "Speeding up logistic model tree induction". In: *Knowledge Discovery in Databases: PKDD 2005.* Springer, 2005, pp. 675–683.
